# Advances in Emerging Catalytic Materials for the Conversion of Carbon Dioxide

**DOI:** 10.3390/ma16237309

**Published:** 2023-11-24

**Authors:** Bingyue Sun, Bingquan Wang, Rui Wang

**Affiliations:** 1 School of Environmental Science and Engineering, Shandong University, No. 72 Seaside Road, Qingdao 266237, China; 2 School of Chemistry and Molecular Engineering, Qingdao University of Science and Technology, Qingdao 266042, China

**Keywords:** CO_2_ capture, CO_2_ utilization, CO_2_ catalytic conversion, ionic liquid, single-atom catalyst, dendritic mesoporous silica nanomaterial, N-heterocyclic carbene

## Abstract

The use of fossil fuels leads to significant CO_2_ emissions, thus highlighting the importance for investigating the utilization of CO_2_ for generating high-value chemical products toward achieving the dual-carbon goal. CO_2_ can be efficiently used in synthesizing valuable organic compounds through C-C, C-O, C-H, and C-N bond construction, with reduction technologies effectively converting CO_2_ to organic carbon sources. Therefore, the research in developing environmentally friendly catalysts for efficient and renewable CO_2_ conversion holds great importance. New materials for catalytic conversion include zeolites, activated carbon, graphene, metal-organic frameworks (MOFs), covalent organic frameworks (COFs), ionic liquids, semiconducting photocatalysts, single-atom catalysts (SACs), and dendritic mesoporous silica nanoparticles (DMSNs). The proper research and use of these materials can aid in the quest to reduce carbon emissions and mitigate climate change. This Review focuses on the utilization of single-atom catalysts (SACs), ionic liquids (ILs), dendritic mesoporous silica nanoparticles (DMSNs), and carbene-metal catalytic systems in CO_2_ conversion. The potential for new materials in catalyzing the conversion of CO_2_ is examined by analyzing various common chemical carbon sequestration methods, ultimately providing possible research directions for effective solutions to climate and environmental pollution problems. On the basis of the high reaction rate and high treatment efficiency of the catalyst for the catalytic conversion of CO_2_, the Review focuses on the simpler and more economical synthesis method of the catalyst itself and the wider application prospects.

## 1. Introduction

Owing to increased human activities since the Industrial Revolution, such as burning fossil fuels (including coal, oil, and natural gas), deforestation, and land use alteration, carbon dioxide (CO_2_) emissions have drastically increased, surpassing 400 ppm and projected to triple by 2040 [1]. CO_2_ is a major greenhouse gas that has a significant impact on Earth’s temperature and climate. Increasing CO_2_ emissions contribute to increased global warming and climate change, with a greenhouse effect that triggers extreme weather events, such as frequent heatwaves, droughts, floods, and sea-level rise.

**Figure 1:**
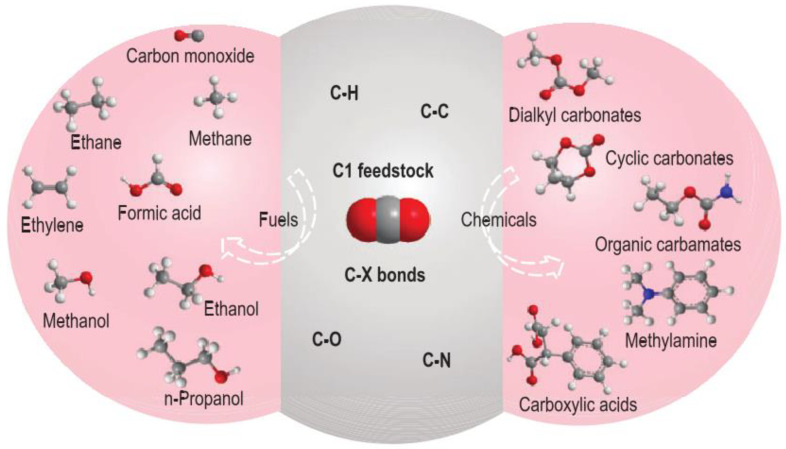
Value-added fuels and chemicals produced from CO_2_ transformation through the construction of various C-X bonds [8].

The conversion of CO_2_ to a wide range of value-added products by electrocatalysis, photocatalysis, or thermocatalysis provides a practical carbon-neutral or even carbon-negative idea in the energy utilization process. In particular, photocatalysis has been considered as an intensely promising green technology for environmental remediation and clean energy production [2] and photocatalytic technology that can accelerate the CO_2_ reduction reaction induced by sunlight [3]. Therefore, it is considered as a potentially important way to reduce CO_2_ emissions and form valuable products at the same time. CO_2_ can be used as a feedstock for the further synthesis of fuels and chemicals through the formation of various chemical bonds, such as C-H, C-C, C-O, and C-N bonds [4]. The Value-added fuels and chemicals produced from CO_2_ transformation through the construction of various C-X bonds (Figure 1). CO_2_ reduction is an important route for CO_2_ utilization, where CO_2_ is converted to many platform compounds, such as hydrocarbons, acids, and alcohols, through the construction of C-H bonds. In addition, the utilization of CO_2_ as one of the reactants to synthesize valuable products is an emerging strategy for CO_2_ conversion. When CO_2_ is used in carboxylation reactions, C-C bonds can be formed to produce valuable products, such as carboxylic acids and organic carbonates. Meanwhile, cycloaddition reactions of CO_2_ with substrates, such as epoxides, aziridines, or propargylamines, can form C-O or C-N bonds and produce cyclic carbonates and oxazolidinone derivatives. Technical term abbreviations are explained upon their first use. According to known scientific reports, one of the most environmentally friendly and efficient atom economical strategies is the chemical coupling of CO_2_ and epoxides into cyclic carbonates under mild conditions owing to their unique properties, such as significant polarity and high boiling point, enabling the industrial synthesis of very important compounds [5].

However, because CO_2_ is a linear molecule, high thermodynamic stability and chemical inertness make it difficult to activate, and the kinetic difficulties of CO_2_ reduction arise mainly from the first step in the conversion of CO_2_ to produce the CO intermediate, which has an equilibrium potential of −1.9 V (vs. SHE), and a large energy difference between the lowest unoccupied molecular orbitals of the CO_2_ molecule and the highest occupied molecular orbitals (≈13.7 Ev) [5]. Photo- and electrocatalysts face several challenges, including the inefficient use of visible light, inadequate CO_2_ adsorption, poor product selectivity, and low yield of reduced products during CO_2_ reduction, which hinder further technological development. Furthermore, the stable CO_2_ structure poses a significant obstacle to the CO_2_ reduction reaction. And during the reduction reaction of CO_2_, a high overpotential is required in the first step of the conversion from CO_2_ to CO, so the competitive and, usually, kinetically favorable reduction from H_2_O to H_2_ must be prohibited. The catalytic reduction of CO_2_ has been studied extensively in recent years, including through thermochemical, electrochemical, photochemical, and biochemical pathways. Meanwhile, adsorption-based gas separation processes have been widely investigated for CO_2_ capture and separation owing to their energy-efficient, environmentally friendly, and highly efficient properties [6]. Various porous adsorbents, such as activated carbon, activated alumina, carbon nanotubes, zeolites, graphene-based porous materials, and metal-organic frameworks (MOFs), have been widely investigated for the capture and separation of CO_2_ [7].

The development of electrocatalysts possessing high selectivity and activity is the key to the efficient catalytic conversion of CO_2_ and promotion of the sustainable development of society. Therefore, in this paper, we analyze the recent progress in “research hotspot” catalysts for CO_2_ catalytic reduction, summarize the preparation strategies and application directions of the CO_2_ catalytic reduction process, and emphasize the excellent electrocatalytic activities of SACs and molecular catalysts for CO_2_ reduction. In addition, an outlook is provided for studying the CO_2_ reduction mechanism and exploring the catalyst structure–activity relationship and for the prospects of CO_2_ reduction and challenges to be faced (Figure 1).

## 2. Emerging Catalytic Materials

### 2.1. Ionic Liquids (ILs)

Ionic liquids (ILs) are substances that are made up entirely of ions, are liquids at or near room temperature, and contain organic cations and inorganic or organic anions. As a new type of green solvent, ILs have unique advantages, such as high thermal stability, designability, feasible design capabilities, as well as excellent solubility. At the same time, according to the needs of practical applications, ILs can be synthesized by changing the combination of anions and cations to change the polarity, hydrophobicity, viscosity, and other properties; further purposefully synthesize functionalized ILs, which can be used to replace traditional volatile polar solvents; and demonstrate a vast range of application possibilities.

In the traditional IL-catalyzed CO_2_ conversion process, the low CO_2_ capacity and high viscosity of ILs limit the movement of charged particles, thus limiting the mass-transfer movement and the conductivity of the solution, which hinder the CO_2_ diffusion and low current density in the conversion process. In terms of application improvement, to solve the problems of high-viscosity ILs, new functional ILs can be designed and used to further improve the solubility of CO_2_, reduce the viscosity of ILs, and optimize volumetric parameters, such as electrolyte pH values. In addition, water or non-aqueous solvents with higher CO_2_ solubilities can be used to form electrolyte solutions and combined with ILs for photocatalytic CO_2_ reduction [9], and some commonly used non-aqueous solvents are methanol, dimethylsulfoxide, acryl carbonate, tetrahydrofuran, acetonitrile, etc.

The ILs studied to date have different roles in the catalytic reduction reaction of CO_2_ because of their different ionic compositions. The cations are mainly dominated by imidazole cations [10], which can complex with anionic radicals (CO_2_^−^) and lower the reaction potential. In addition, cations inhibit the competitive hydrogen precipitation reaction by forming a monomolecular layer on the electrode [9]. The dominant anions include halogen ions and inorganic-acid ions, while imidazolium-based ILs facilitate formate generation by enabling charge transfer between anions and semiconductors, thereby reducing the activation energy barrier for CO_2_ [11]. As for research applications, in the photo- and electrocatalytic reductions of CO_2_ with the participation of ILs, ILs are mainly present in the reaction medium, playing the roles for promoting charge transfer and improving the adsorption and activation of CO_2_ [12]. In certain studies, ILs were loaded on the surface of photo/electrocatalysts as co-catalysts to promote the adsorption and activation of CO_2_, ultimately boosting the reaction conversion and product selectivity [13].

#### 2.1.1. IL-Based Electrolytes Improve CO_2_ Catalytic Conversion Efficiency

Properties, including structure, conductivity, viscosity, CO_2_ solubility, polarity, and stability, enable ILs in the reaction medium to play the roles for facilitating charge transfer, increasing CO_2_ solubility, and inhibiting hydrogen precipitation reactions. Therefore, when ILs are used as electrolytes, the cations, anions, functional groups, and even the length of alkyl chains of ILs should be considered. By far, imidazolium-based ionic liquids are the most used class of ILs. Imidazolium-based ionic liquids have been extensively researched for CO_2_ catalytic conversion and utilization owing to their significant trapping capacity for CO_2_ [14].

The disadvantages of conventional ILs, such as low CO_2_ capacity and high viscosity, limit the movement of charged particles, thus limiting the mass-transfer motion and the conductivity of the solution, which hinder the diffusion of CO_2_ and generate a low current density during the conversion process [15]. To solve the problem of the high viscosity of ILs, in addition to the design and use of novel functionalized ILs to further improve the solubility of CO_2_, the IL viscosity can be reduced, volumetric parameters (such as the pH of the electrolyte) can be optimized [16,17], and water or non-aqueous solvents (which have a higher CO_2_ solubility) can be used and together with the ILs, constitute the electrolyte solution for photo-electrocatalytic CO_2_ reduction [18]. Some commonly used non-aqueous solvents are methanol, dimethyl sulfoxide, acryl carbonate, tetrahydrofuran, and acetonitrile [18,19].

**Figure 2:**
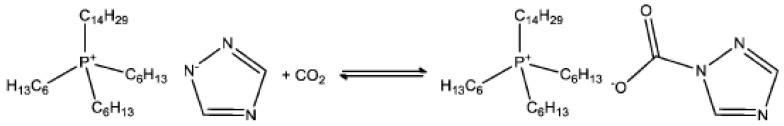
Addition of CO_2_ to [P66614] [124Triz], showing binding of CO_2_ to the triazolide anion [20].

Because of their unique tunable properties, including negligible vapor pressures and high thermal stabilities, ILs offer an opportunity for addressing this challenge to develop CO_2_ capture systems. A great deal of effort has been focused on experimental and theoretical studies on the physical absorption of CO_2_ in ILs. The enthalpy of the CO_2_ physical absorption by ILs is about 20 kJ mol^−1^, indicating that only a quarter of the energy is required to release the physically absorbed CO_2_ from ILs in the regeneration step relative to the amine solution method. The conventional physical adsorption of CO_2_ in the same ionic liquid or in ionic liquids where chemisorption is impossible (e.g., [P66614] [NTf_2_]) results in a substantial increase in the overpotential, leading to a reduction reaction for which CO is the only product and is generated at a low rate (Figure 2). A recent subgroup of ILs called “superbasic ILs” (SBILs) are being researched for gas capture applications owing to their higher CO_2_ capacities and low viscosity increases compared to those of conventional ILs. SBILs consist of quaternary phosphonium/ammonium-based cations paired with a superbasic anion, such as imidazolide, triazolide, or phenoxide. SBILs absorb equimolar amounts of CO_2_ through the chemical interaction of CO_2_ with anions and the physical absorption of CO_2_ in the free space of the solution, thus solving the problem of low solubility, and have been widely used for CO_2_ capture [9]. Nathan and colleagues utilized ultra-strong basic ionic liquids to electrochemically reduce CO_2_ to formate and synthesized gases at a low overpotential to achieve a new low-energy pathway for catalytic CO_2_ reduction by ultra-strong basic ionic solutions and further applications in CO_2_ catalytic reduction [20].

**Figure 3:**
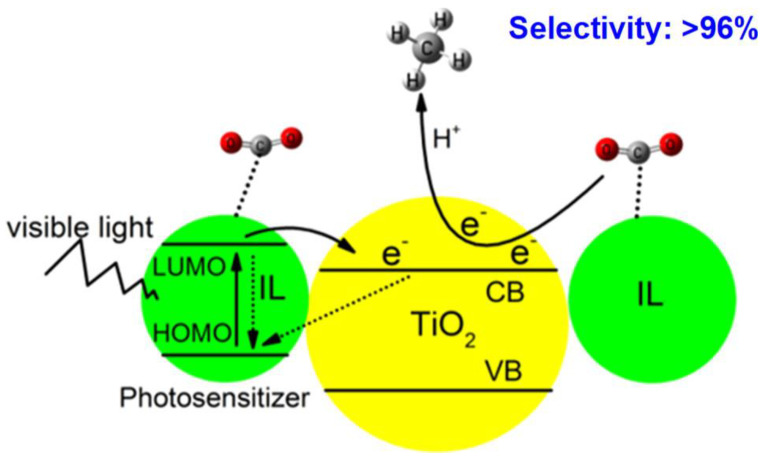
Proposed CO_2_ photoreduction process under visible light irradiation, using the IL as the photosensitizer and CO_2_ absorbent [22].

In the study of the photothermal catalytic reduction of CO_2_, imidazolium-based ionic liquids are deemed appropriate as CO_2_ trapping media owing to their capacity to activate CO_2_ and convert it to chemicals. Figure 3 reveals the Proposed CO_2_ photoreduction process under visible light irradiation, using the IL as the photosensitizer and CO_2_ absorbent. By taking advantage of the key roles of ILs in inhibiting H_2_O reduction and improving the ethanol yield, Li et al. constructed a Cu_2_O/g-C_3_N_4_ heterojunction and used it for the photocatalytic reduction from CO_2_ to ethanol. The ethanol yield of Cu_2_O/g-C_3_N_4_ reached 0.71 mmol g^−1^ h^−1^, which was 1.89 times higher than that of photocatalysis and 7.05 times higher than that of thermocatalysis, and significantly reduced the reaction temperature for the conversion from CO_2_ to ethanol under photo- and thermocatalysis [21].

#### 2.1.2. Ionic Liquids as Co-Catalysts

ILs can activate thermodynamically stable CO_2_, capture activated CO_2_ intermediates, and accelerate their transfer to the reaction interface used as a co-catalyst for the catalytic reduction of CO_2_, which facilitates the reduction from CO_2_ to CO at electrodes, significantly lowers the reaction overpotential, and inhibits the hydrogenolysis reaction (HER) [23]. Additionally, a vast quantity of ILs can adjust the microenvironment on the catalyst surface and modify the ECR selectivity through the inclusion of diverse intermediates in the direct electrochemical reduction of CO_2_ [24]. In fact, the excellent co-catalytic performance of ILs is mainly because the intermediates formed by ILs and CO_2_ push electrons toward the anions in ILs, leading to stronger nucleophilic activation. Feng et al. exploited the co-catalytic role played by ILs at the metal–catalyst interface to regulate the interaction between CO_2_ molecules and active sites, promote the CO_2_ supply near the electrode/electrolyte interface, and address the CO_2_ transport for the efficient direct electrochemical reduction of CO_2_ [25].

Cadena et al. [26] suggested that using cationic imidazole groups could enhance CO_2_ solubility, arguing that the dominant factor controlling the solubility of CO_2_ in ILs is the type of anion in alkyl imidazole group-based ILs. Chen et al. improved the CO_2_ absorption of the system by designing conjugated anionic complexes in ionic liquids to form ILs with visible-light-absorbing CO_2_ complexes that catalyze the reduction from CO_2_ to CH_4_ under visible-light irradiation [22].

#### 2.1.3. Progress and Prospects of ILs in CO_2_ Catalytic Reduction Studies

ILs have been widely used in the catalytic conversion of CO_2_ because of their high CO_2_ solubility and excellent electrolyte properties, mainly acting as trapping agents and co-catalysts. Various types of ILs and IL-based mixtures have been investigated, with imidazole-based ILs being the most extensively researched and utilized. The designability of ILs enables the integration of functional ILs for CO_2_ conversion to optimize conversion pathways. This approach has significant promise for creating co-catalyst environments in other electrocatalytic reactions. Furthermore, the replacement of traditional volatile solvents with ILs can prevent fugitive solvent losses, thereby mitigating atmospheric pollution from organic matter [27]. Thus, as designable solvents, ILs are potential options for energy- and cost-efficient CO_2_ capture. Finally, practical separation processes having higher CO_2_ selectivities for other gases were also considered. Ether-based functionalized ILs were designed for the selective separation of CO_2_ from natural gas and biogas, with an average increase in CO_2_-CH_4_ selectivity of 50% compared to those of unfunctionalized analogs [28].

During the use of ILs, it is necessary to consider the potential environmental risks they may pose. ILs have the potential to hinder the growth of flora and fauna. Furthermore, certain IL structures possess the capacity to persist as pollutants owing to their resistance to degradation processes [29]. Therefore, it is crucial to identify the hazards associated with the presence of ILs in the environment. In addition, the preparation of ionic liquid-based membranes by laminating ILs with polymeric membranes is another good alternative to avoid the high viscosity of ionic liquids and may lead to a significant improvement in gas separation.

### 2.2. Single-Atom Catalysts for CO_2_ Catalytic Conversion

**Figure 4:**
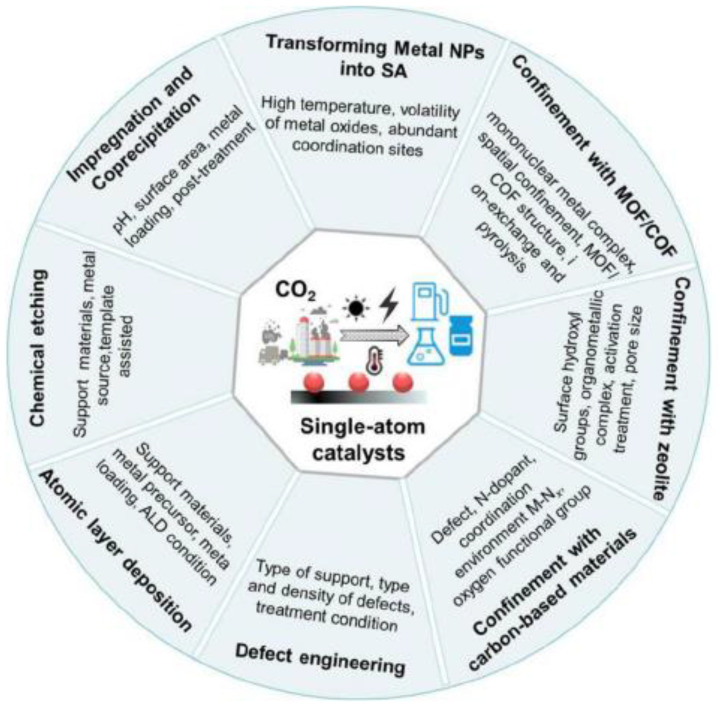
Schematic diagram of synthetic methods and key parameters for SACs [31].

Single-atom catalysts (SACs), which have metal atoms well dispersed on solid carrier surfaces, show outstanding performance in CO_2_ electroreduction reactions because of their robust interactions with single-atom carriers and remarkable catalytic efficiency. Furthermore, the maximum metal utilization is achieved in these catalysts. SACs have aroused significant interest in heterogeneous catalysis in recent years because of their high catalytic selectivity and tunable activity in various chemical reactions [30]. Single-atom catalysts have gained popularity in research owing to their ability to minimize catalyst costs by maximizing the metal dispersion and atom utilization efficiency. The schematic diagram of synthetic methods and key parameters for SACs (Figure 4). However, SACs exhibit particle agglomeration, low metal loadings, and difficulty in large-scale manufacturing. In addition, molecular catalysts, another type of SAC, consist of ligand molecules linked to metal ions and exhibit high activities for CO_2_ reduction and possess metal–nitrogen (M-N) active centers similar to those of metal–N(M-N)-C(M-N-C) SACs, which are highly effective for CO_2_ reduction because their well-defined active sites have tunable spatial and electronic properties.

#### 2.2.1. Design Principles of SACs in the Catalytic Conversion of CO_2_

The maximum utilization efficiency of SACs can be achieved through their distinctive electronic properties, atomic dispersion, and ligand releasing. The maximum metal utilization efficiency on SAC surfaces yields a higher catalytic activity per surface atom than that on nanoparticle (NP) surfaces [32]. The large specific surface area and complex shape of nanoparticle materials can fulfill the requirements of a wide range of applications [33]. In addition, strong interactions between metal sites and carriers offer the potential to finely adjust the intrinsic electronic configuration of metal centers by modifying the coordination environment. At the same time, isolated metal sites can greatly influence the properties of their neighboring atoms.

Various methods have been used for the design of SACs, and different strategies have emerged for the efficient synthesis of SACs, such as impregnation and co-precipitation, defect engineering, atomic layer deposition, conversion from metal nanoparticles to SACs, spatially confined domains (MOFs, COFs, zeolites, carbon-based materials, etc.), and chemical etching. The practical utilization of single atoms requires not only high activity but also high stability and high density [34]. Physical deposition (e.g., ALD) is used to dope single atoms into a suitable substrate material and offers the ability to precisely control the size and distribution of particles on the substrate surface via sequential and self-limiting surface reactions. This technique offers a high degree of stability, the ability to uniformly deposit films layer by layer on the surfaces of complex carriers, and the ability to direct the growth rates of deposited materials. Atomic layer deposition (ALD) has been applied to anchor isolated Pt atoms to graphene nanosheets [35]. Cheng and colleagues synthesized a range of size-variable Pt species derived from isolated single atoms and clusters and loaded on N-doped graphene, enabling the precise control of the Pt dimensions, modulation of coordination environments, and fabrication of different Pt electronic structures for highly selective CO_2_ reduction [31].

Catalysts produced by the ALD approach are restricted by low metal loadings, which are attributed to the high equipment cost and low yield for scalable production. The low metal loading and particle aggregation during the catalyst preparation compound the difficulty in utilizing ALD. Furthermore, the large-scale synthesis and control of stable SACs and clusters remain challenging.

#### 2.2.2. Application of SACs in the Catalytic Conversion of CO_2_

Isolated metal atoms can be loaded onto various carriers, and a single metal site has multiple spatial configurations and coordination environments, which significantly improve the activity and stability of SACs. Meanwhile, SACs have low overpotentials, which are conducive for promoting the inhibition of the hydrogen precipitation reaction during the catalytic reduction of CO_2_. This makes SACs ideal for electrocatalytic, photocatalytic, and thermocatalytic reactions, which surpass the applications of conventional nanoparticle-based catalysts. Furthermore, SACs have enormous potential in the research and development for converting CO_2_ to high-value-added products [36].

SACs for the electrocatalytic conversion from CO_2_ to CO have been prepared by N- and S-doping or by enhancing the interactions of isolated atoms with the catalyst. For metal–nitrogen–carbon (M-N-C) catalysts possessing an asymmetric coordination structure, the N atoms have a higher electronegativity, leading to a greater concentration of electrons in their vicinity. This effectively reduces the electron cloud density around the C atoms, which facilitates the adsorption of molecules, such as O_2_, and, thus, promotes the catalytic reaction. By comparing various active sites, Xu and colleagues proposed that asymmetrically coordinated M-N-C active sites further promoted their catalytic performance in electrocatalytic ORR, CO_2_RR, HER, OER, and NRR [37].

The main reactions in thermocatalytic CO_2_ conversion are CO_2_ hydrogenation and conversion to methane. However, the high stabilities of CO_2_ and CH_4_ usually require high reaction temperatures (600–1000 °C), which tend to lead to rapid deactivation of the catalyst due to carbon and char accumulation. Thermocatalysis offers higher efficiencies and yields than electrocatalytic and photocatalytic reductions. Wang et al. synthesized Pt-Fe-NG catalysts by atomic layer deposition (ALD) through the introduction of Fe atom-bridged Pt nanoparticles and N-doped graphene carriers, which enabled the catalysts to exhibit optimal *OH adsorption and suitable metal–carrier interactions. The Fe-bridged atoms enhanced the interaction between the Pt nanoparticles and graphene while lowering the energy barrier for *OH protonation, resulting in the excellent ORR activity and stability of the Pt-Fe-NG catalysts [38].

The photocatalytic conversion from CO_2_ to CH_4_, HCOOH, C_2_H_4_, and CH_3_OH can be achieved under mild conditions, with the reaction being manageable. However, a large amount of energy is consumed during the reaction, resulting in a low photocatalytic conversion efficiency. For specific products, the photocatalytic CO_2_ yield and structure–activity relationships were improved for CO_2_RR on noble and non-precious metal SACs. Dong et al. prepared Pt single-atom-anchored amorphous ZrO_2_ nanowires (PtSA/ZrO_2_) as an efficient photocatalyst for CO_2_ reduction. The co-catalyst exhibited an excellent CO yield (16.61 mmol/g/h) and selectivity (97.6%) for improving the efficiency of photocatalytic CO_2_ reduction [39]. The design strategy based on anchoring single atoms to amorphous nanosubstrates significantly improves the activity, selectivity, and durability of photocatalysts. This approach will encourage the development of advanced photocatalysts.

#### 2.2.3. Perspectives of SACs in the Catalytic Conversion of CO_2_

Single atoms show potential for CO_2_ catalytic conversion research but are susceptible to particle aggregation. There are still some challenges for the development of SACs with high loadings on a large scale and the maintenance of the unique structures of active sites tightly connected to the metal centers and carriers during the preparation process. Many substrates have been explored for the development of SACs. Carbon carriers, such as carbon nanotubes, graphene oxide, metal–organic skeleton-derived nanocarbon, and carbon nitride, offer several advantages for electronic applications. These benefits include high electrical conductivity, specific stability, favorable surface chemistry, and low cost [40,41].

### 2.3. Dendritic Mesoporous Silica Nanomaterials

Nanomaterials possess outstanding physical, chemical, and electrical properties that differentiate these materials from conventional materials [42]. Recently, dendritic mesoporous silica nanomaterials (DMSNs) have attracted attention owing to their excellent performance and modification mechanisms. Dendritic mesoporous silicon oxide nanoparticles are a class of silica materials possessing a central radially three-dimensional mesoporous structure [43], and the name “dendritic mesoporous” refers to the similarity of the pore structure to a three-dimensional distribution of dendrites. DMSNs have a special central radially mesoporous structure, high specific surface area, rich morphology [44], excellent solvent dispersion, and good biocompatibility [45], which make DMSNs a research hotspot for CO_2_ capture and catalytic conversion.

Additional nanoparticles are incorporated utilizing DMSNs’ high specific surface areas and pore volumes. The three-dimensional structure of DMSNs significantly enhances the accessibility of the active sites, resulting in a further improvement in catalytic activity. In addition, DMSN materials can be synthesized quickly and easily, within a matter of hours [46]. The nanocomposites can be functionalized and modified by doping with active elements [47]. Therefore, DMSNs have high application value in the catalytic conversion from CO_2_ to organic compounds.

#### 2.3.1. DMSNs in the Catalytic Conversion of CO_2_

DMSNs, loaded with carrier ionic liquids, serve as optimal catalyst carriers for generating noble-metal-based catalysts that exhibit high accessibility to active sites and exceptional catalytic activity. Additionally, these carriers are eco-friendly [48]. KCC-1/IL/HPW nanocatalysts exhibited excellent catalytic activity in the reaction under mild conditions for the synthesis of cyclic sulfur carbonyl carbonates from carbon dioxide and epoxide, using fibrous silica nanocarbonate as a carrier loaded with high-specific-surface-area heteropolyacid-based ionic liquids (KCC-1/IL/HPW) [49]. The high catalytic activity, easy recovery from the reaction mixture by filtration, and ability to be reused several times without any significant loss of performance are additional environmental attributes of this catalytic system [50]. Regarding the formation of five-membered cyclic carbonates from CO_2_ and epoxides, the most important aspect of the published studies is that they are increasingly focused on environmental friendliness and general sustainability. The fixation of CO_2_ to chemicals has also attracted much research attention because it extends the use of higher-value materials. The reaction of CO_2_ with epoxides for the easy commercial production of many useful chemicals is one of the methods that promotes general sustainability [51].

To solve the problem that CO_2_ cannot be catalyzed alone in the photocatalytic reduction of CO_2_, He et al. synthesized Sn (IV)-doped PrVO_4_ nanoparticle catalysts ((SnD)-loaded PrVO_4_ nanoparticles; (PrVO_4_/SnD)) by an in situ method. The catalyst is potentially highly dynamic in stabilizing CO_2_ for the production of 2-oxazolidinone and benzimidazole, with a certain degree of reproducibility and high efficiency [52].

Owing to the large specific surface areas and multiple catalytically active sites of DMSNs, ionic gels can be introduced at the interface of DMSNs [53]. Asadi et al. prepared FeNi3@DMSN-IG-Pd by functionalizing core–shell FeNi_3_@DMSNs in ionic gels and loading them uniformly with Pd nanoparticles without aggregation. The prepared products can efficiently catalyze the multi-component coupling of CO_2_, thereby facilitating the synthesis of β-carbonyl carbamates that are recyclable and reusable [54].

#### 2.3.2. Potential of Dendritic Mesoporous Silica Nanoparticles in Catalytic Reduction of CO_2_

Dendritic mesoporous silica nanoparticles exhibit distinctive chemical compositions and morphological structures. Their synthesis methodology and the factors influencing their performance have been exhaustively explored, leading to outstanding reuse capabilities. However, their hindered industrial application is mostly attributed to the complexity in synthesizing numerous kinds of dendritic mesoporous silica nanoparticles in addition to several influencing factors and low yields. Meanwhile, the special advantage of dendritic mesoporous silica nanoparticles is their unique pore structure, but the thickness [55,56], shape, and length of the pores are still more difficult to precisely control in current studies. Therefore, the more accurate regulation of the pore structures of dendritic mesoporous silica nanoparticles will become the focus of attention to meet the demands of customized applications.

### 2.4. N-Heterocyclic Carbene Catalytic Reduction of CO_2_

N-heterocyclic carbenes are neutral compounds [57] consisting of a carbene carbon center adjacent to a layered sandstone nitrogen atom, which is usually present in the ring structure. N-heterocyclic carbenes possess high coordination and electron-donating capacities [58], exhibit stable structural properties, and are inexpensive and easy to prepare [59,60]. During the synthesis process, NHCs can coordinate with Cu, Ag, Ru, Pd, and other metals [61] to form stable complexes [61,62], which play the role of CO_2_ catalytic activation; thus, NHCs have attracted greater attention in the field of CO_2_ functionalization in recent years.

#### 2.4.1. NHCs and Metals Form a Variety of Efficient Catalysts

NHCs have become common ligands in organometallic chemistry because strong σ-electron-donating NHC ligands offer the opportunity to modulate the reactivity and selectivity of transition-metal catalysts. Taking copper as an example of a widespread and eco-friendly metal, NHCs can combine with it to form different efficient catalysts for carbon dioxide conversion. Of those, NHC–copper serves as a feasible catalyst for the carbon dioxide reduction reactions of organoboron compounds. The complexes undergo nucleophilic addition with carbon dioxide to produce the corresponding lipid compounds, thus chemically immobilizing carbon dioxide molecules. The reaction of NHC–copper catalysts with diboron compounds to generate reactive intermediates is the most critical step in the whole carbon sequestration process. In 2016, Butcher and colleagues demonstrated the catalytic boron carboxylation reaction of olefins by redox-neutral copper catalysts. This work expands the range of substrates and enables further opportunities for applications of NHC–copper [63]. Despite the advantages of homogeneous Cu-NHC complexes, such as high activity and selectivity, their poor recyclability and reusability limit their use in practical applications. In addition, residual metals and their products may cause severe problems in the synthesis of biologically active and functional substrates [64]. In recent years, there have been studies on the reaction of CO_2_ with epoxides to produce polycarbonates and/or cyclic carbonates. Cyclic carbonates are used in excellent aprotic polar solvents, as electrolytes in secondary batteries, as precursors of polycarbonates, and in biomedical applications. These products have high boiling points and, therefore, have high commercial value as solvents [65].

#### 2.4.2. NHC Catalytic Reduction of CO_2_

**Figure 5:**
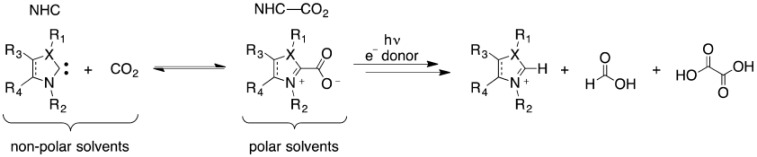
Stability and photochemical reduction of NHC-CO_2_ [56].

Although metal catalysts are commonly used, conventional metal catalyst systems present issues, such as high costs, low selectivity, and poor durability. With the development of the green chemistry concept, the study of metal-free catalysts has received increasing attention from researchers in recent years. NHCs themselves are strongly nucleophilic and can undergo strong nucleophilic coordination with carbon dioxide, forming stable amphiphilic N-heterocyclic 2-carboxylate (NHC-CO_2_). In this instance, NHCs can produce various carbene-carboxylates, which can participate in various chemical transformations and are the key to the reductive fixation of CO_2_. Therefore, as nonmetallic catalysts, N-heterocyclic carbenes have excellent application prospects in the field of CO_2_ functionalization. Figure 5 shows NHC−CO_2_ models with various substituent effects.

In the N-heterocyclic carbene (NHC)-catalyzed reduction–functionalization from CO_2_ to formamide, Zhou et al. proposed that NHCs act as neither CO_2_ nor silane activators but catalyze reactions by forming new species possessing higher catalytic activities [66]. In 2012, Cantat and colleagues reported the NHC-catalyzed reduction–functionalization of CO_2_ to formamide, using silane as a reducing agent and an amine as a functionalization reagent [67]. Ratanasak et al. used triphenylborane to smoothly catalyze the N-methylation of N-methylaniline with CO_2_ (1 atm) and PhSiH_3_ at 30 °C in the absence of a solvent. Other catalysts that have been reported to date usually require higher temperatures for the N-methylation of amines with CO_2_ and hydrogen-containing silanes. Therefore, the catalytic system composed of BPh_3_ and PhSiH_3_ exhibits significant catalytic activity in CO_2_ reduction fixation. This finding holds great research value and is expected to drive future advancements in the field [68].

#### 2.4.3. Limitations of NHC Catalytic Reduction of CO_2_

In addition, NHCs can contain an unlimited number of additional functionalities, which grant this unique ligand class a noteworthy edge in the synthesis of customized homogeneous catalysts and open the door to a plethora of potential applications [58]. However, NHCs still exist as fewer species and have more restrictions for effective metal coordination, making it crucial to select NHCs possessing high activities and stabilities while also expanding the number of metal-loaded species and developing NHC–metal catalysts possessing higher activities for NHC applications. Meanwhile, most present research focuses on homogeneous catalytic systems involving NHCs. NHC catalysts are difficult to recycle and do not satisfy the current concept of green chemistry.

## 3. Conclusions and Perspectives

**Figure 6:**
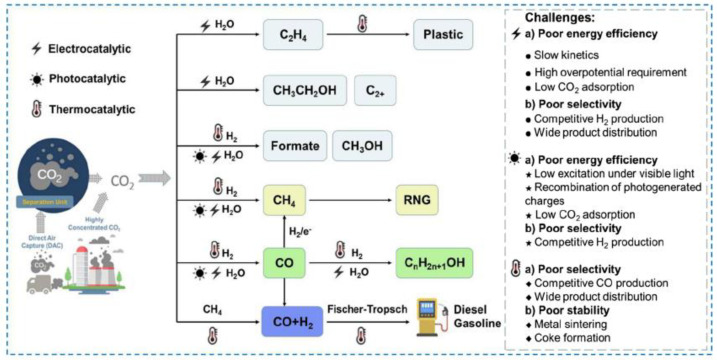
Applications of various routes and major challenges in catalytic CO_2_ transformation processes via electro-, photo-, and thermocatalytic technologies [31].

The efficient and gentle conversion of CO_2_, the most important greenhouse gas, to high-value-added carbon-based chemicals and fuels and the promotion of CO_2_ resource utilization offer a promising strategy for reducing CO_2_ emissions and alleviating society’s reliance on fossil fuels. Figure 6 shows the applications of various routes and major challenges in catalytic CO_2_ transformation processes via electro-, photo-, and thermocatalytic technologies. CO_2_ can be effectively used to construct C-C, C-O, C-H, and C-N bonds to synthesize high-value-added organic compounds, such as polyols, dimethyl carbonate (DMC), formic acid (FA), cyclic carbonate (CCS), polycarbonate (PC), and methane, which is a highly efficient, environmentally friendly, and potentially useful method for improving the carbon cycle and, therefore, can be utilized to reduce CO_2_ emissions via CO_2_ reduction technology. CO_2_ reduction technology can be applied to effectively utilize and further convert CO_2_ to other organic carbon sources. However, the linear structure and high thermal stability of CO_2_ make direct utilization more challenging, and traditional CO_2_ reduction technology must be used at high temperatures and high pressures, which requires expensive and safe equipment as well as consumes significant amounts of energy. The potential for developing CO_2_ catalytic reduction methods using catalysts under mild conditions is significant. These catalysts demonstrate great promise for promoting CO_2_ reduction under mild conditions. This Review focuses on the use of emergent catalysts, such as single-atom catalysts (SACs), ionic liquids (ILs), dendritic mesoporous silica nanoparticles (DMSNs), and carbene–metal composites, for CO_2_ conversion. Although there have been abundant catalyst research advances for the conversion of CO_2_, the lack of practical applications is still a major concern. Future studies may consider the following possibilities:

(1) Thermochemical, photochemical, and electrochemical carbon dioxide reduction technologies are presently being investigated, and there are still problems, such as an insufficient understanding of the reaction mechanism of CO_2_ catalytic conversion, low carbon dioxide conversion rates, low yields, and poor reaction stabilities;
(2) The recyclability and reusability of catalysts are desirable, following the principle of green chemistry, which requires low-cost, simple, and highly stable catalytic recycling processes;
(3) For synthesizing composite catalysts, there are problems, such as complicated synthesis processes, too many influencing factors, low yields, and poor material utilization rates, which considerably hinder the industrial application of catalysts;
(4) At present, the application of a single technology for the catalytic activation of CO_2_ remains a challenge. The combined use of light, electricity, heat, plasma, and other measures seems to work in some cases, but the underlying mechanisms must still be revealed. Toward that goal, the development of catalysts for the efficient conversion of CO_2_ under mild reaction conditions is esteemed.

In summary, the development of more efficient, inexpensive, stable catalysts that can be produced at a large scale, regenerated with low energy consumption, and operate under mild conditions for the catalytic reduction of CO_2_ is an important direction for future research on CO_2_ conversion. The design and development of high-performance catalytic materials, by taking into account both thermodynamics and kinetics, as well as the enhancement of mass-transfer processes while improving the adsorption capacity will be conducive to the low-cost, high-efficiency, and rapid capture of CO_2_.

## Data Availability

No new data was created in this study.
